# Drug Delivery Using Novel Biological and Synthetic Materials

**DOI:** 10.1155/2015/682578

**Published:** 2015-11-10

**Authors:** Yoshihiro Ito, Eiry Kobatake, Jun Li, Seung-Wuk Lee

**Affiliations:** ^1^Nano Medical Engineering Laboratory, RIKEN, Wako-shi, Saitama 351-0198, Japan; ^2^Department of Environmental Chemistry and Engineering, Graduate School of Interdisciplinary Science and Engineering, Tokyo Institute of Technology, Yokohama-shi, Kanagawa 226-8502, Japan; ^3^Department of Biomedical Engineering, Faculty of Engineering, National University of Singapore, Singapore 117602; ^4^Department of Bioengineering, University of California, Berkeley, Berkeley, CA 94720, USA

Drug delivery is important for administering a pharmaceutical compound to efficiently achieve a therapeutic effect in humans or animals. For these systems novel biological and synthetic materials have been developed. One is controlled release of the active agent with a predetermined time course such as constant, oscillating, declining continuously, or even pulsatile mode. In other systems, materials have been designed with targeting or pathology-responsive functions. Recent development of biomaterials has expended the new possibilities of drug delivery systems. Included are implantable inorganic materials, nonbiodegradable and biodegradable polymers, biological materials, and the hybrid biomaterials derived from synthetic and natural macromolecules.

The articles contained in the present issue are original papers describing current and expected challenges along with novel biological and synthetic materials for drug delivery. The issue comprises the description of materials for delivery of nucleic acids and proteins.

Since the discovery of RNA interference (RNAi) and the achievement of gene silencing by synthetic small interfering RNAs (siRNAs), siRNA has become established as a new tool for silencing target genes. siRNAs have, therefore, been widely recognized as novel potential therapeutics. To date, there has been considerable effort to develop siRNA therapeutics for treating viral infections and cancers. However, naked siRNA is readily degraded by nucleases and siRNAs are too large and hydrophilic to cross cell membranes. Therefore the developments of delivery method or appropriate gene carriers are required for siRNA therapeutic applications.

In the paper by T. Nishimura et al. entitled “Amylose-Based Cationic Star Polymers for siRNA Delivery,” the authors describe a new siRNA delivery system using a cationic glyco-star polymer. They prepared spermine-modified 8-arm amylose star polymer by chemoenzymatic methods and characterized the spherical complex with siRNA. It was concluded that the amylose-based star polymers are a promising nanoplatform for glyco biomaterials.

P. He et al. in the paper entitled “Low-Molecular-Weight Polyethyleneimine Grafted Polythiophene for Efficient siRNA Delivery” investigated a new class of synthetic polymer carrier that consisted of polythiophene. Their concept underlying the design of these copolymers was that hydrophobicity and rigidity of polythiophenes should enhance the transport of siRNA across the cell membrane and endosomal membrane. They showed their developed copolymers serve as novel, low cell toxicity, and efficient siRNA delivery systems.

For not only siRNA delivery but also gene delivery, S. Rao et al. compared the utility of aviral gene delivery vectors in their paper “The Comparative Utility of Viromer RED and Lipofectamine for Transient Gene Introduction into Glial Cells.” Cationic lipid (Lipofectamine) lipoplex or polyethylenimine (Viromer RED) polyplex technologies were examined in cell lines and primary glial cells for their transfection efficiencies, gene expression levels, and toxicity. The authors found the transfection efficiencies of polyplex and lipoplex agents were comparable in a limited, yet similar, transfection setting, with or without serum across a number of cell types.

In order to deliver proteins, A. Haider et al. reported a ceramics/polymer hybrid in their paper entitled “BMP-2 Grafted nHA/PLGA Hybrid Nanofiber Scaffold Stimulates Osteoblastic Cells Growth.” They used bone morphogenetic protein (BMP) on the nanofiber to stimulate the growth of osteoblastic cells. They suggested that BMP-g-nHA/PLGA hybrid nanofiber scaffold could be used as a nanodrug carrier for the controlled and targeted delivery of BMP-2, which will open new possibilities for enhancing bone tissue regeneration and will help in the treatment of various bone-related diseases in the future.

C. Suttinont et al. investigated delivery of another growth factor in their paper “Delivery of bFGF for Tissue Engineering by Tethering to the ECM.” They chose basic fibroblast growth factor (bFGF) as the growth factor and developed the growth factor-tethered extracellular matrix (ECM). The designed ECM was comprised of a stable structural unit and included the well-known cell adhesive RGD peptide as an active functional unit. They showed the effectiveness of the designed protein.

We hope that this special issue would shed light on major developments in the area of new drug carriers and attract attention by the scientific community to pursue further investigations leading to the rapid implementation of these materials in clinical applications.

